# 810 Synthetic Platelet-Mimetics with Gentamicin: Outcomes in Deep Partial-Thickness Burns

**DOI:** 10.1093/jbcr/irac012.358

**Published:** 2022-03-23

**Authors:** Franklin Valdera, Kristo Nuutila, Laura E Cooper, David E Varon, Jennifer Cooley, Javier Chapa, Rodney K Chan, Anders H Carlsson

**Affiliations:** Department of Surgery, Brooke Army Medical Center, San Antonio, Texas; U.S. Army Institute of Surgical Research, San Antonio, Texas; Department of Surgery, University of Maryland Medical Center, San Antonio, Texas; University of Michigan Medical School, San Antonio, Texas; Department of Surgery, William Beaumont Army Medical Center, El Paso, Texas; Division of Combat Wound Repair, U.S. Army Institute of Surgical Research, San Antonio, Texas; US Army Institute of Surgical Research, San Antonio, Texas; The Metis Foundation, San Antonio, Texas

## Abstract

**Introduction:**

Infection and prolonged inflammation in deep partial-thickness burns can lead to inadequate healing. In addition to their role in hemostasis, activated platelets also contain granules with anti-inflammatory properties which may impact wound healing. However, portability and storage of platelets remain a challenge. Synthetic platelet-mimetics (SPM) avoid these difficulties, but like natural platelets, SPM have vesicles that can transport bioactive agents, such as antibiotics. We sought to evaluate wound healing outcomes in deep partial-thickness burns treated topically with SPM.

**Methods:**

A total of 30 circular, deep partial-thickness burns were created on the dorsum of 2 porcine models. Each wound measured 5cm in diameter and was standardized using a thermocoupled burn device. Sets of six wounds were randomized into five groups: SPM, gentamicin alone, SPM with gentamicin, a vehicle control (saline), or dry gauze (the standard of care, SOC). Additionally, two separate 5cm diameter circular areas were demarcated to serve as normal skin controls. Wounds were assessed at post-burn days 3, 7, 14, 21, 28, 60 and 90 with a variety of non-invasive imaging and punch biopsies. Treatments were re-applied at each assessment. The primary outcome was the amount of wound re-epithelialization, measured histologically, at post-burn day 28. Secondary outcomes consisted of the percentage of wound contraction, amount of superficial blood flow, and mean bacterial load.

**Results:**

The amount of wound re-epithelialization in wounds treated with SPM was 96% whereas those treated with the SOC measured only slightly less at 92% (p = 0.56). The percentage of wound contraction was 39% in the SPM with gentamicin group, while in the SOC group contraction was higher at 45% (p = 0.20). Moreover, superficial blood flow in the SPM group was measured to be 168% of normal skin controls, while wounds in the SPM with gentamicin group were slightly lower at 160%, and in wounds treated with the SOC blood flow was 110% of normal skin controls (p = 0.27). The mean bacterial load in each group was measured at post-burn day 3 and notably consisted of bacterial load being the lowest in wounds treated with gentamicin alone at 17/100, meanwhile in wounds treated with the SOC or those in the SPM with gentamicin group, mean bacterial loads were significantly higher at 43/100 and 47/100, respectively (p = 0.02).

**Conclusions:**

SPM applied to deep partial-thickness burns did not significantly improve measured outcomes over the SOC. Although numerical improvements were shown in all measured outcomes, no significant differences were noted. The theoretical and observed potential for SPM to improve wound healing deserves additional evaluation in larger preclinical studies.

